# Pulmonary and Medullary Tuberculosis: An Uncommon Cause of Evans Syndrome in Adults

**DOI:** 10.7759/cureus.74378

**Published:** 2024-11-25

**Authors:** Halima Hadri, Hanane Delsa, Yasmine Zouiten, Mouna Khmou, Said Benchekroun

**Affiliations:** 1 Hematology, Cheikh Khalifa International University Hospital, Mohammed VI University of Sciences and Health, Casablanca, MAR; 2 Hematology and Oncology, Research Unit, Mohammed VI Center for Research and Innovation, Rabat, MAR; 3 Gastroenterology and Hepatology, Cheikh Khalifa International University Hospital, Mohammed VI University of Sciences and Health, Casablanca, MAR; 4 Research Unit, Mohammed VI Center for Research and Innovation, Rabat, MAR; 5 Pathology, National Institute of Oncology, Rabat, MAR

**Keywords:** antitubercular treatment, corticosteroid, evans syndrome, pancytopenia, tuberculosis

## Abstract

Evans syndrome (ES) is a rare syndrome characterised by the association of autoimmune idiopathic hemolytic anemia (AIHA) with immune thrombocytopenia (ITP) and, less commonly, autoimmune neutropenia (AIN). ES may be primary or secondary to some aetiology, including, exceptionally, tuberculosis. We describe a case of association between pulmonary and medullary tuberculosis and Evans syndrome with an effective response to antitubercular treatment and corticosteroids.

## Introduction

Evans syndrome (ES) is defined by the simultaneous or sequential association with autoimmune idiopathic hemolytic anemia (AIHA), immune thrombocytopenia (ITP), and, less commonly, autoimmune neutropenia (AIN) [1]. First described by Evans in 1951, it's a rare syndrome [2]. Diagnosing ES requires excluding differential diagnoses and determining whether ES is primary or secondary. The combination of Evans syndrome and tuberculosis (TB) is extremely rare and requires a very comprehensive evaluation to exclude other etiologies of ES. We report the case of a patient diagnosed with pulmonary and medullary tuberculosis whose pancytopenia was associated with Evans syndrome during evolution, with good outcomes on antitubercular treatment and corticosteroids.

## Case presentation

A 72-year-old female with a 15-year history of insulin-dependent diabetes and a previous diagnosis of peritoneal tuberculosis, treated with antitubercular therapy 6 years ago, was admitted to the pulmonology department for a pulmonary infection. The infection developed over several days and was associated with a dry cough, purulent sputum, fever and general deterioration. Subsequently, she required intensive care. Vitals on presentation revealed blood pressure 100/70 mmHg, pulse 127/min, respiratory rate 25/min, SpO_2_ 88%, and temperature 39.4°C. On physical examination, she was pale, with edema and petechiae of the lower limbs, without palpable lymph nodes or hepatosplenomegaly. Chest auscultation revealed diminished breath sounds in both lung bases. 

The patient's complete blood count (CBC) showed anemia, with hemoglobin (Hb) at 5.6 g/dL, mean corpuscular volume (MCV) at 84.4 fL, mean corpuscular hemoglobin concentration (MCHC) at 34.1 g/dL, white blood cell (WBC) count at 1260/mm³, neutrophils at 460/mm³, lymphocytes at 500/mm³, and platelet count at 22000/mm³. The hemostasis test was normal, with no evidence of cytolysis or cholestasis. C-reactive protein (CRP) was elevated at 336 mg/L (Table 1). The nasopharyngeal swab for severe acute respiratory syndrome coronavirus 2 (SARS-CoV-2) was negative.

**Table 1:** Results of the patient's laboratory assessment on admission and during treatment with corticosteroids W: Week

Weeks (W)	Admission	Before corticosteroid treatment	W1	W2	W3	W24	Reference values
Hb (g/dL)	5.6	5.2	8.2	9.6	10.8	11.1	12-16 g/dL
Neutrophils (/mm^3^)	460	520	960	1050	2010	2100	2000-7000/mm^3^
Platelet (/mm^3^)	22000	16000	72000	98000	125000	200000	150000-450000/mm^3^
C-reactive protein (mg/L)	336	-	160	-	65	8	<10 mg/L
Procalcitonin (ng/mL)	1.9	-	-	-	0.3	-	<0.5 ng/mL
Indirect bilirubin (IB)	8	57	-	-	7	-	2-7 mg/L

**Figure 1:**
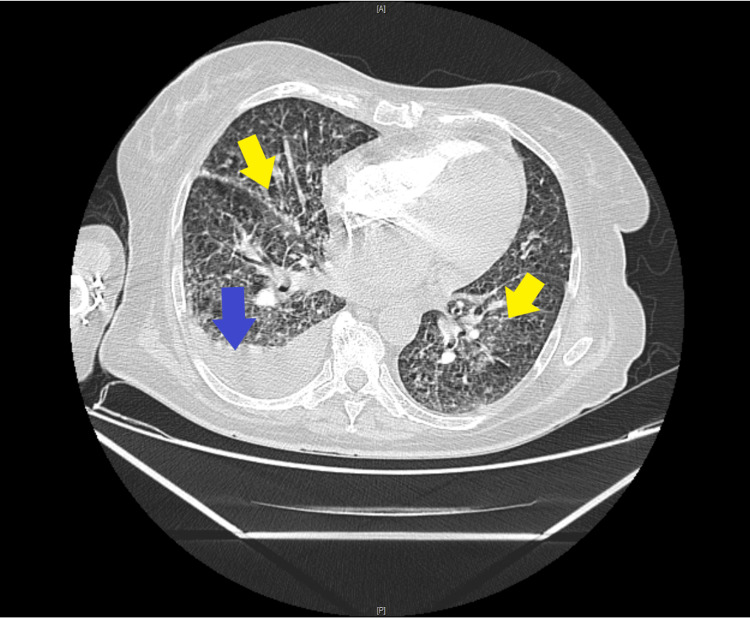
Axial computed tomography images of thorax showing bilateral nodules and micronodules with a miliary appearance (yellow arrow) and a moderate pleural effusion on the right side (blue arrow), suggestive of tuberculosis

A computed tomography (CT) scan of the chest revealed bilateral nodules and micronodules with a miliary appearance and a moderate pleural effusion on the right side, suggesting tuberculosis (Figure 1). Sputum testing for tuberculosis (TB) was positive. The myelogram showed the presence of all cell lines without cytological abnormalities, and a bone marrow biopsy confirmed the diagnosis of medullary tuberculosis.

**Figure 2:**
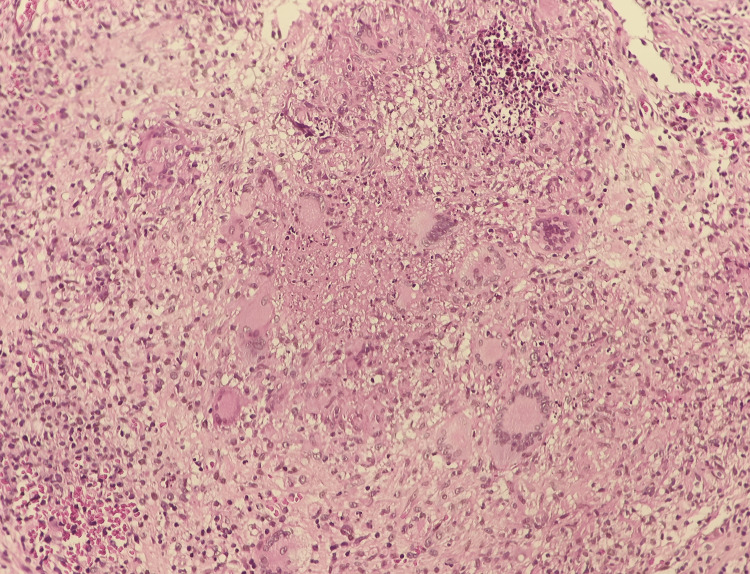
Hematoxylin and eosin staining of paraffin-embedded tissue section (original magnification ×200): Image showing epithelioid and giant-cell granulomatous formations, centered around a focus of caseous necrosis

The Quantiferon-TB and sputum testing for TB (sputum smears and cultures) were positive. The bone marrow aspiration showed the presence of all cell lines without cytological abnormalities, and bone marrow biopsy confirmed the diagnosis of medullary tuberculosis (Figure 2).

The patient had started antitubercular treatment with isoniazid, rifampin, pyrazinamide, and ethambutol. One month after starting treatment, the patient still had persistent pancytopenia and was admitted to the emergency department due to extreme fatigue. Her CBC showed Hb at 5.2 g/dL, a WBC of 1400/mm³, neutrophils at 520/mm³, lymphocytes at 510/mm³, and a platelet count of 16,000/mm³. Her lactic dehydrogenase (LDH) level was 467 U/L, indirect bilirubin (IB) was 57 mg/L, and the reticulocyte count was 145,000/mm³. The direct antiglobulin test (DAT) was positive for IgG without complement (C3). Electrolytes and creatinine were within normal limits (Table 2).

**Table 2:** Follow-up laboratory results after one month of antitubercular treatment.

Laboratory examination	Results	Reference range
Hemoglobin (Hb) g/dL	5.2	12.5-16.5
Mean corpuscular volume (MCV) (fL)	92	83-97
White blood cell (WBC) Count (/mm^3^)	1400	4000-10000
Neutrophil count (/mm^3^)	520	2000-7000
Lymphocyte count (/mm^3^)	520	1500-4000
Platelet count (/mm^3^)	16000	150000-450000
Reticulocyte count (/mm^3^)	145000	25000-75000
Total bilirubin (mg/L)	60	2-12
Indirect bilirubin (mg/L)	57	2-7
Lactate dehydrogenase(LDH) (U/L)	313	155-230
Direct antiglobulin test (DAT)	Positive (IgG)	Negative
Urea (g/L)	0.50	0.15-0.45
Creatinine (mg/L)	6.9	6-12
Sodium (Na^+^) (mEq/L)	134	136-145
Potassium (K^+^) (mEq/L)	3.6	3.5-5.1
Calcium (Ca^++^) (mEq/L)	89	85-101
Chlorure (Cl^-^) (mEq/L)	93	98-107
Ferritin (ng/mL)	318	30-230

The extended etiological work-up, including viral serologies, hepatitis, HIV, Epstein Barr virus (EBV), cytomegalovirus (CMV), parvovirus B19, influenza, and immunological work-up, was negative. Corticosteroid based on prednisone at a dose of 1.5 mg/kg was started with gradual improvements in hematological parameters until normalization (Table 1). Blood tests remained normal with slow tapering over six months.

## Discussion

Evans syndrome (ES) is characterized by the simultaneous or sequential occurrence of autoimmune idiopathic hemolytic anemia (AIHA) and immune thrombocytopenia (ITP), with autoimmune neutropenia (AIN) being a less frequent component [1]. It was first described by Evans in 1951 [2]. ES is a rare disorder with an annual incidence of 1.8/million person-years, as reported in a Danish study with 242 patients followed over four decades. The median age of ES is 58.5 [55.9-61] years [3]. In over 80% of cases, ES is chronic (lasting more than 1 year) and is slightly more common in women. Interestingly, in 27-50% of cases, ES is combined with other diseases, notably hematological malignancies and adult systemic lupus erythematosus [3].

AIHA is characterized by the presence of anemia (hemoglobin less than 12g/dl in males and 11g/dl in females) associated with a reticulocyte count >120 000/mm3 and indicators of hemolysis such as an increase in lactate dehydrogenase (LDH), a decrease in haptoglobin and an increase in indirect bilirubin (IB). In addition, a positive direct antiglobulin test (DAT) for IgG, with or without complement (C3d), suggests autoimmunity, provided cold agglutinins are excluded in the context of Evans syndrome [1].

ITP is defined by a platelet count <150 000/mm3; it is often lower and must be unrelated to liver disease (cirrhosis and portal hypertension), splenomegaly (sequestration thrombocytopenia), drug-induced thrombocytopenia, bone marrow deficiencies such as myelodysplastic syndromes and hematological malignancies [4]. The biological diagnosis is made using the monoclonal antibody platelet immobilization assay (MAIPA). This test has a sensitivity and specificity of up to 81% and 98%, respectively. This test should be reserved for difficult cases and is not always recommended for routine use [4,5].

AIN is suspected when the neutrophil count is <2000 /mm3 and other causes of neutropenia have been excluded (drug-induced neutropenia; viral infections such as cytomegalovirus (CMV), Epstein-Barr virus (EBV), human immunodeficiency virus (HIV), parvovirus B19 and influenza; myelodysplastic syndrome or leukemia). Diagnosing ES requires excluding differential diagnoses and determining whether ES is primary or secondary. The recommended investigations to find secondary causes of ES are listed in Table 2 [1].

**Table 3:** Recommended tests for secondary causes of Evans syndrome *HCV: Hepatitis C virus; **HBV: Hepatitis B virus [1]

Recommended tests for secondary causes of ES
Viral tests (HIV, HCV*, HBV**, EBV, CMV, parvovirus B19); serum protein electrophoresis, protein immunofixation, and immunoglobulin concentrations; antinuclear antibodies and anti-dsDNA antibodies; lupus anticoagulant assay and antiphospholipid antibodies; blood smear; circulating lymphocyte phenotyping; flow cytometry for the detection of clones in paroxysmal nocturnal hemoglobinuria; bone marrow aspiration and karyotype; bone marrow biopsy; CT scan of the chest, abdomen and pelvis.

Once these investigations have been carried out, the various etiologies of ES must be ruled out, such as hematological malignancy, autoimmune disorders (AID), primary immunodeficiencies (PID) in children, various viral infections (hepatitis C virus (HCV), Epstein Barr virus (EBV), cytomegalovirus (CMV) and varicella virus (VZV) [1], and more recently SARS-CoV-2 has been reported as a potential cause of ES [6]. Forms associated with pregnancy should also be excluded [7]. 27-50% of ES cases are secondary to other diseases [3] and need recognition at diagnosis, as mortality associated with secondary ES is close to 50% [8]. Only a few cases of ES in association with tuberculosis (TB) have been described in the literature [9-13].

Tuberculosis is an infectious bacterial disease caused by *Mycobacterium tuberculosis*. The main site of involvement is the lungs, but the disease can be extrapulmonary and affect many organs. Bone marrow infiltration by TB is manifested in patients by the complete or incomplete association of anemia, thrombocytopenia, and neutropenia [14]. Another cause of anemia in TB is erythrocyte destruction by antibodies produced by lymphocytes in response to the tubercular pathogen; it is also possible that these antibodies destroy other blood cells [13,14].

Based on the lung involvement and pancytopenia, the patient was diagnosed with pulmonary and bone marrow tuberculosis. Anti-tuberculosis treatment was administered, but the worsening of pancytopenia and the tests performed revealed Evans syndrome, probably secondary to tuberculosis, as there was no other identifiable cause. The patient was treated with a prednisone-based corticosteroid therapy (11.5 mg/kg/day) for 4 weeks in addition to the anti-tuberculosis treatment, with a good result.

Corticosteroids are first-line treatment, with 1mg/kg prednisone daily for 3-4 weeks, tapered rapidly over 1 week in the case of ES thrombocytopenia [15] and slowly over 6 months in the case of ES anemia [16]. In ES, increasing the dose of prednisone to 1.5 mg/kg for a period of 3-4 weeks may be suggested. This is to avoid the side effects of corticosteroids, particularly the worsening of TB [12]. Transfusion Support and intravenous immunoglobulins (IVIG) can be used with corticosteroids in the first-line therapy of ES [1].

In unresponsive or corticosteroid-refractory patients, second-line therapy should be started rapidly. We can use rituximab with an initial response rate of 60% and long-term remissions of 30% [17]; splenectomy with complete responses in both isolated ITP and isolated AIHA of 66% [15] and 40% respectively [16]; immunosuppressants such as cyclophosphamide, azathioprine, cyclosporin or mycophenolate and hematopoietic stem cell transplantation can be proposed [1].

## Conclusions

Evans syndrome in association with tuberculosis is a rare and serious situation. ES is difficult to diagnose, and other causes must be ruled out before tuberculosis is suggested as the etiology of the ES. This is an extremely rare situation and the use of corticosteroids in tuberculosis remains a challenge.
